# SMSaúde: Design, Development, and Implementation of a Remote/Mobile Patient Management System to Improve Retention in Care for HIV/AIDS and Tuberculosis Patients

**DOI:** 10.2196/mhealth.3854

**Published:** 2015-03-09

**Authors:** José António Nhavoto, Åke Grönlund, Walter Ponce Chaquilla

**Affiliations:** ^1^InformaticsSchool of BusinessÖrebro UniversityÖrebroSweden; ^2^InformaticsDepartment of Mathematics and InformaticsEduardo Mondlane UniversityMaputoMozambique; ^3^Elizabeth Glaser Pediatric AIDS Foundation (EGPAF)MaputoMozambique

**Keywords:** mobile health, text messaging, SMS system, patient management, design science research, Mozambique

## Abstract

**Background:**

The widespread and low cost of mobile phones and the convenience of short message service (SMS) text messaging suggest potential suitability for use with alternative strategies for supporting retention in care and adherence to the treatment of various chronic diseases, such as HIV and tuberculosis (TB). Despite the growing body of literature reporting positive outcomes of SMS text message-based communication with patients, there is yet very little research about the integration of communication technologies and electronic medical records or electronic patient tracking systems.

**Objective:**

To design, develop, and implement an integrated mobile phone text messaging system used to follow up with patients with HIV and TB in treatment in Mozambique.

**Methods:**

Following the design science research methodology, we developed a Web-based system that provides support to patients. A case study involving three health care sites in Mozambique was a basis for discussing design issues for this kind of system. We used brainstorming techniques to solicit usability requirements, focus group meetings to discuss and define system architecture, and prototyping to test in real environments and to improve the system.

**Results:**

We found six sets of system requirements that need to be addressed for success: data collection, telecommunication costs, privacy and data security, text message content, connectivity, and system scalability. A text messaging system was designed and implemented in three health facilities. These sites feed data into a central data repository, which can be used for analysis of operations and decision support. Based on the treatment schedule, the system automatically sent SMS text message appointment reminders, medication reminders, as well as motivational and educational messages to patients enrolled in antiretroviral therapy and TB treatment programs.

**Conclusions:**

We successfully defined the requirements for, designed, and implemented a mobile phone text messaging system to support HIV and TB treatments. Implementation of this system could improve patients’ self-management skills and strengthen communication between patients and health care providers.

##  Introduction

### The HIV and Tuberculosis Problem

HIV/AIDS and tuberculosis (TB) are major public health problems in many developing countries around the world [1]. Globally, in 2012, an estimated 35.5 million people were living with HIV and some 2.3 million people were newly infected [2]. Of the global burden, Sub-Saharan Africa had 25 million people living with HIV and nearly 1.6 million new HIV infections [2]. In 2013, an estimated 9.0 million people developed TB and 1.5 million died from the disease, 360,000 of whom were HIV-positive [3]. In Mozambique, in 2012, 1.6 million were living with HIV and there were 120,000 new infections [2]. In 2013, nearly 140,000 developed TB and close to 18,000 died from the disease in Mozambique [3].

One major problem with HIV/AIDS and TB treatment is ensuring patients pursue their treatment, including medication and medical checkups until completion. Hence, there is a need to improve the adherence and retention in care. While there may be many reasons for the lack of endurance, there may be ways to improve completion of treatment programs by maintaining better contact (communication) between doctors and patients.

### Mobile Interventions

Mobile phone technology has the potential to serve as a strategic intervention medium to improve patient management [4]. Due to the widespread use and low cost of this technology, it pervades all age groups and many cultures and socioeconomic backgrounds, including in developing countries. It allows communication across geographic boundaries and reaches people directly where they are located [4].

Mobile phone short message service (SMS) text messaging is well suited for supporting self-management and improvement of patients' self-efficacy skills through, for instance, medication reminders [5-10] and motivational text messages [8,11]. But there is also a need for interactive program management by reacting promptly and effectively to deviations from program plans. This may include operational decisions for short-term management of patient retention at individual care centers, tactical decisions such as reconfiguring the messaging based on performance, and strategic decisions such as data collection or preservation.

Mobile phones integrated with electronic health records (EMR)—in the context of HIV and TB programs called electronic patient tracking systems (EPTS)—could have the potential to empower the health care system and improve patients’ welfare, as well as help patients improve self-management of their disease. For instance, mobile phones could be used to transmit data collected from a patient’s mobile phone to a remote system [12,13] where data is processed and accessible for physicians. This data could then be used to send medical recommendations to patients [14-16]. For example, mobile phones were incorporated into a central terminal for personal health examination, where a system referred to as mobile health examination launched on the phone (M-HELP) could generate electronic health records and send them to physicians via email or multimedia messages [17].

Effects of the use of mobile phone text messaging on antiretroviral therapy (ART) adherence have been investigated in a number of studies. A study in Kenya examined adherence and found that weekly text messaging enhanced ART adherence and improved suppression of viral load [18]. In Brazil, SMS text messages were able to help Brazilian women living with HIV/AIDS to remain adherent to ART for a period of 4 months [19]. Daily text messaging was found to be a feasible and acceptable way to remind HIV-positive youth with poor adherence to ART to take their medications, and there was a significant increase in adherence rates, postintervention [20]. In a study with participants from Cameroon and Kenya, text messaging was found to have a significant effect on adherence to ART [21]. A systematic review by Nglazi and colleagues [22] found only four studies that compared the outcomes of the SMS text message intervention group with controls for promoting adherence to TB treatment. One example is a study in South Africa, where SMS text message reminders were equally effective compared to the more resource-requiring directly observed therapy, short-course (DOTS) strategy [23]. In another study, SMS text message reminders were utilized when patients were delayed in opening their medication bottles, and increased TB cure and smear conversion rates were reported compared to DOTS [24]. In Kenya, SMS text message reminders increased rates of clinic attendance on scheduled days compared to standard care [8]. However, not all SMS text message interventions yield positive results. For instance, in Argentina SMS text message intervention did not significantly improve adherence to tuberculosis treatment compared to self-administration [25]. A study by Mills and colleagues [26] found 14 interventions using daily and weekly text messages as treatment supports, alarms, and counselling. The study found that treatment supports with enhanced standard of care (SOC) and weekly text messages were significantly better than basic SOC [26]. A similar Cochrane review reported that SMS reminders improved outcomes—adherence to medication or to treatment in 49% (24/60) of the studies, appointment attendance in 18% (11/60) of the studies, and nonattendance rates decreased in 18% (11/60) of the studies [27]. In summary, there are many positive reports, but as not all cases are successful there appears to be an implementation factor—implementation can be done in many ways and the above Cochrane review suggests that only some of them are successful.

### Justification for the Study

Despite the growing body of literature documenting the outcomes of text messaging-based intervention and, in some cases, its integration with EPTS, it has become important to document not only outcomes, but also development procedures. To this end, it is of great importance to report the engineering behind such positive or negative effects of the use of mobile phone text messaging-based technologies. In the literature, few studies are available that routinely provide sufficiently detailed descriptions about the requirements—design, development, and implementation processes—of these interventions [28], which are particularly important as not all studies show positive results. Ngabo and colleagues [29] described requirements for designing and implementing a mobile phone-based communication system aimed at monitoring pregnancy and reducing bottlenecks in communication associated with maternal and newborn deaths [29]. Consolvo and colleagues [14] reported their experiences with developing a prototype mobile phone app and presented four design requirements for technology that encourages physical activity: (1) give users proper credit for activities, (2) provide personal awareness of activity level, (3) support social influence, and (4) consider the practical constraints of users’ lifestyles [14]. What is yet largely lacking in the research literature, however, are studies analyzing all the practical technical decisions taken in developing the integrated system. For this reason, this paper focuses especially on the design, development, and implementation of a text messaging system integrated into the electronic medical records of HIV/AIDS and TB patients to help them efficiently manage their treatment.

### Case Description

In 2008, Absolute Return for Kids (ARK)—a United Kingdom-based international organization whose purpose is to transform children’s lives—in partnership with the Ministry of Health in Mozambique, launched a 5-year program in five health facilities in the Maputo province aiming to put in place a sustainable model of care to keep HIV-positive parents, caregivers, and children alive. In 2012, ARK started pilot-testing an SMS text messaging study (SMSaúde) to encourage patients to return to the health facilities for treatment, sending text messages about appointment dates and the need to take medication regularly. This system was pilot-tested over 1 year and 3 months. Toward the end of 2012, ARK planned to scale up the project to 16 more health centers, but there was a need to improve the system. The pilot system lacked some features, such as automatic reply to patients’ messages and an interface for the visualization of (patient’s) data. Also, the administration of the system was complicated, laborious, and error-prone as it was distributed to each heath care site.

In this paper, we show how we took part in helping ARK to overcome the barriers facing the plan to improve the system and scale up the project to reach another 16 health centers in the Gaza province, Mozambique.

### Study Objective

The study objective of SMSaúde was to evaluate the use of mobile phone text messaging to improve retention in HIV and TB care in Mozambique. This paper focuses on three key activities—defining the requirements, design and development, and implementation—in the creation of a prototype of SMSaúde aiming to facilitate communication between patients and the health care system, and to improve patient retention to HIV/AIDS and TB treatments.

## Methods

### Research Framework

The research framework applied in this study is design science research (DSR) [30]. DSR involves a rigorous process to design artifacts to solve observed problems, to make research contributions, to evaluate the designs, and to communicate the results to appropriate audiences. The design-science paradigm draws on the sciences of the artificial [31] and is constructive to its nature—“The process of constructing and exercising innovative IT artifacts enable design-science researchers to understand the problem addressed by the artifact and the feasibility of their approach to its solution” [30].

The project followed the design science research methodology (DSRM) [32] and the proposed solution was operationalized in a prototype developed by the authors of this paper. To implement the prototype, three health care centers within the ARK organization were selected for the case study. DSRM comprises six steps: (1) problem identification and motivation, (2) objectives of a solution, (3) design and development, (4) demonstration, (5) evaluation, and (6) communication [32]. DSRM focuses on solving real-world problems through creation and evaluation of information technology (IT) artifacts [30]. DSRM is, therefore, appropriate in this research since the situation required intervention in real-world operations. In this paper, we focused on the first five steps of DSRM.

### Problem Identification and Motivation

This step requires a definition of the specific research problem and justification of the value of the solution [32]. As part of this exercise, it is necessary to provide a problem definition that will be used to develop an artifact that can effectively provide a solution [32]. As part of justifying the value of the solution, it is important to motivate the researcher and the audience of the research so as to pursue the solution and accept the results [32].

To investigate the current system, the first author of this paper (JAN) had four meetings with ARK staff. The output of these meetings was a document describing a new approach. The approach proposed to redesign the SMSaúde system attempting to meet specific requirements and adding more functions that would allow ARK to use the system in another province with 16 health facilities.

### Objective of a Solution

The aim of this step is “to infer the objectives of a solution from the problem definition and knowledge of what is possible and feasible” [27].

Solution objectives were formulated through discussions between two ARK’s staff (project coordinator and project director) and the first author of this paper. Several iterations were made in the process of formulating the objectives for the new systems. During these iterations, the researcher first presented and explained the initial version of the objectives. Subsequently, the project coordinator from ARK reviewed the solution objectives. The feedback provided by the project coordinator was used by the researchers to reformulate the solution objectives. Again, this presentation and review cycle continued until satisfactory solution objectives were achieved.

### Design and Development

This activity creates the artifacts—constructs, models, methods, or instantiations—and includes determining the artifacts’ desired functionalities and their architectures [32].

A broad set of usability requirements was identified during two brainstorming design sessions between the researchers and two medical doctors. The doctors were researchers with strong clinical backgrounds and substantial experience in health care and transversal understanding of the problems faced by patients and health workers. During the first session, the medical doctors outlined the requirements that they anticipated the system should offer. These requirements were then documented and elaborated in more detail as new ideas were exchanged within the design team (two medical doctors and two computer experts). All sessions were recorded, transcribed, and analyzed. The design principles of the system were derived from these sessions.

The general architecture of the system was analyzed and refined in discussion with the development team. The development team was composed of three undergraduate students in informatics and lead by the first author. Next, the team went through an interactive process that frequently switched between programming steps and discussion meetings where the prototype was tested. Mainly, the discussion meetings focused on informing rapid prototyping via identification of implications for technology design and enhancement.

The development team and the two medical doctors assessed the responses from patients in the current system, which were stored in a Microsoft SQL server, to identify patterns that could be used to define relevant answers. The output was a list of possible automatic SMS text message replies.

### Demonstration

In this step, it is necessary to demonstrate the use of the artifact to solve instances of the problem [32]. The activity of demonstration could be effectuated in experimentation, simulation, case study, or other appropriate activity [32].

The SMSaúde system was pilot-tested in five health facilities: Machava 2, Matola 2, Matola 1, Ndlavela and Namaacha. All five centers provide both routine HIV treatment and support to patients in treatment for HIV/AIDS, and standard TB chemotherapy. In the pilot study, 750 patients were selected using the following inclusion/exclusion criteria: 1) currently in first line of antiretroviral treatment, 2) aged 18 years or older, 3) have basic literacy skills in Portuguese, 4) own a cell phone, and 5) not be part of other ongoing research.

### Evaluation

Here, the artifact is observed and measured to find out how well it supports a solution to the problem [32]. Evaluation “involves comparing the objectives of a solution to actual observed results from use of the artifact in the demonstration” [32].

We evaluated the SMSaúde system in terms of functionality, completeness, consistency, accuracy, performance, reliability, and usability quality attributes [30].

### Ethical Approval

The design of this study was approved from an ethical perspective by the Institutional Bioethics Committee on Health of Faculty of Medicine and Maputo Central Hospital (CIBS FM&HCM), Maputo, Mozambique (study code CIBS FM&HCM/18/2013).

## Results

### Problem Identification and Motivation

When planning for the new system, a number of problems were encountered. There was no concise documentation of the current system requirements, or of how the system was designed. Cost of operation and maintenance was high and plans for scaling up implied signing a new deal with the designer of the system. Administration was complicated—because the system was decentralized, each time there was a problem with the system the administrator had to travel long distances to solve the issue. Operation of the system made a single person indispensable—if the computer operator was absent from work and others did not know the initiation sequence of system components, the system would not be operational. There was no automatic reply function, something that would allow responses to text messages from patients. There was also no Web-based user interface for management and visualization of information (eg, statistics of incoming and outgoing text messages).

We designed and pilot-tested a new system that we believe solves the aforementioned problems. The next sections map the results of the process followed for design and development, and for implementation of the new system.

### Objectives of the Solution

The objectives finally arrived at were threefold:

1. Design, develop, and implement a text messaging system (SMSaúde) capable of sending four types of SMS text messages—appointment reminders, medication reminders, motivational messages, and educational messages. It should also be capable of receiving text messages from patients and respond to them automatically in a meaningful way.

2. Integrate SMSaúde with EPTS.

3. Integrate SMSaúde across multiple locations with EPTS.

### Design and Development

#### Requirements

##### Overview

Prior to system design, a comprehensive and detailed set of requirements was developed. There were six different requirements, which concerned data collection, telecommunication costs, privacy and data security, the content of text messages, connectivity, and system scalability.

##### Data Collection

It is imperative that the information systems can retrieve data from remote health facilities on a frequent, preferably daily, basis and provide it to the central part of the system, which decision makers use for planning, evaluation, and control of activities. There is also the issue of how often the data is filed in the electronic data system. The team from ARK worked hard to train the data entry clerks so they could enter the data in a very short period of time. The initial agreement was daily data entry with a possible delay of a maximum of 3 days.

##### Telecommunication Costs

In this kind of system there are telecommunication costs involved. It is imperative to be able to make favorable bulk SMS text messaging contracts on the part of the health care provider, but also to be efficient in messaging so as to reduce the traffic. Regarding text messages from patients, there may be some price sensitivity on the part of the patients, but in most cases there should not be that many messages per patient. Another major problem is that in Mozambique, as in most health care systems, costs are measured but not benefits. This means that even if the system leads to more people being cured, the system would still be seen as a cost to the health care system. For innovation to take place, ways to also calculate the benefits must be conceived. To overcome the two problems, ARK signed an agreement with Vodacom, a mobile communications company, that allowed ARK to send free text messages and enabled patients to send free text messages to a short code number.

##### Privacy and Data Security

Data security is a crucial issue in any health care process. We developed an authentication mechanism for clients’ personal computers (PCs) located at the health center. All transactions go in one direction—only the client computers can send data to the server and changes in the server do not affect the data at client computers. The connection between the server and the client computer is made using a Web service.

##### Text Message Content

A set of text messages was developed based upon discussion involving the design team and the medical doctors. Suggestions for message formulations were reviewed and amended during team meetings, resulting in a final set of 32 messages for ART patients and 39 messages for TB patients, which were approved by all involved. In both sets, messages were divided into four categories: appointment reminders, medication reminders, educational messages, and motivational messages. Examples of the types and content of messages are provided in Table 1.

The messages did not include general information about the health status of the patient. This was for privacy reasons, but also because such status information may be difficult to communicate in an unambiguous way. The same goes for additional medical information that may be useful but difficult to communicate accurately.

**Table 1:** Examples of text messages in the four categories.

Category	Example text messages^a^
Appointment	Hello! Your health is above everything. We are waiting for your visit scheduled for xx-xx-xxxx at your Health Center! With health there is joy. Remember that you had a visit scheduled for xx-xx-xxxx. Come to your Health Center. We are waiting for you!
Medication	Dear friend. Do not stop taking the pills otherwise the disease will become stronger and resistant and the retreatment more difficult. Hello! Remember to come and collect your medications to cure TB. You or a relative must always come within the week. Keep on taking them every day without stopping!
Education	Dear friend! It is important that you have a healthy lifestyle, have good nutrition, do not drink alcohol, do not smoke, and get enough rest. Dear friend, your health comes first. Always take your pills daily, at the same time and before eating. It is possible to cure TB!
Motivation	Congratulations! You are now in the maintenance phase. You have completed X weeks. Don't give up! You may feel better but you are NOT better yet. Keep coming! We know you can do it! Dear friend, good job! You are almost done! You have completed XX weeks of treatment and must follow up! We know you can do it. Keep coming!

^a^All messages were translated from Portuguese by the first author.

##### Connectivity

Some Internet access is necessary for a system like this, mainly for data synchronization. Global System for Mobile communications (GSM) modems were used for data communication. As the GSM networks in Mozambique are often unreliable, especially in rural areas, we designed a mechanism of storing data locally and sending it to the main server whenever a connection was established. This way, we were able to in most cases get daily updates, although not necessarily at a predefined time every day.

##### System Scalability

Scalability here means that the system must be able to accept addition of new health centers and more patients. Addition of a health center should not disrupt the normal function of the system. The system was developed with this in mind as there is a need for expansion in the coverage of health centers.

####  System Development

##### Selection of Tools

The system platform selected for the text messaging system was based on the choice of the Web client for the Web-SMS. The Web client provides an interface that allows the professional users on the health care provider side to visualize, navigate, and analyze patient and text message data. We selected three open source-based tools for testing: playSMS, FrontlineSMS, and RapidSMS. The selection of Web-SMS was based on the following criteria: (1) user-friendly interface, (2) ease of interface customization, plug-ins, and functions compatibility, (3) system features (sending, receiving, autoreply, etc), (4) programming languages supported, (5) established community of users and developers, and (6) support of popular SMS text message gateways (eg, Gammu, gnokii, smstools, Clickatell, or Kannel). Based on these criteria, playSMS was eventually selected as it met the majority of the requirements, and a prototype tool was developed. The playSMS system is a flexible, Web-based mobile portal system based on PHP programming language. We installed and preconfigured the Web server environment required to establish playSMS (comprising the Apache Web server, database server MySQL, and PHP).

**Figure 1:**
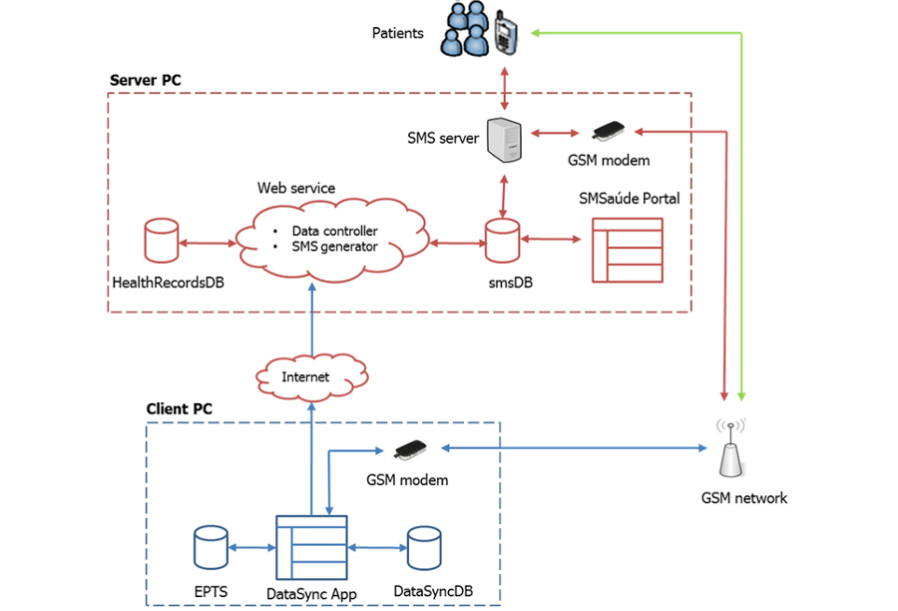
SMSaúde architecture.

To accommodate the requirements, an architecture was developed (see Figure 1). The main elements of this architecture include the following:

1. The databases. There were four databases. The first two databases—Microsoft Access (database system used by the EPTS) and MySQL—were located at the health centers and were used to store patients’ health records. The other two databases—HealthRecordsDB and smsDB—were located at the server side and stored patients’ information received from the health centers, and text messages (outgoing, incoming, and scheduled).

2. Data service (DataSync). DataSync is a service installed in computers located at the health centers. This service connects with the Microsoft Access and MySQL databases and sends filtered data to the central server. Data is sent using a GSM modem.

3. The Web service. The Web service features classes exposed to client computers (client PCs) by using a Web services description language (WSDL)-generating tool using extensible markup language (XML)-based service. Part of these classes are responsible for communicating with DataSync, storing the data in the central database (HealthRecordsDB), and for generating SMS text messages, as well as storing them in the SMS server database (smsDB).

4. The SMS server. The SMS server, as shown in Figure 1, interacts with patients that use their mobile phone set and the SMS messaging service to receive and send messages to the system. At the lowest level, the SMS server interfaces with a GSM modem that sends and receives patients’ SMS text messages through an SMS service provider (mobile operator). Once an SMS text message is received by the modem, the SMS server performs checks on its content and responds to the patient accordingly (automatic reply feature). Every incoming message, after processing by the SMS server, is deleted from the GSM modem, but stored in the smsDB.

The health centers were registered in the remote server. Data from these health centers was sent to the remote server over a secured connection. Safeguarding the privacy, confidentiality, and security of any public health information is an important undertaking. The system only collects data necessary for the server to process and send text messages. The system was operational 24 hours a day, 7 days a week.

##### Communication Between Client PC and Server

The client PC at times must connect to the server and synchronize data. We defined a generic unidirectional gateway. A unidirectional gateway server provides centralized access to required data from the client PC to the server. Thus, integrating any client PC requires only integrating the bridge. In addition, the bridge separates the services that are available remotely from the ones proposed as normal Web services. The gateway server is responsible for formatting the data properly before receiving it from the client PC. Once the data is received from the client PC, its embedded classes are responsible for handling the data.

**Figure 2:**
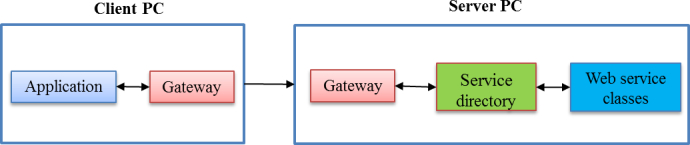
Communication architecture between the client PC and the server.

Figure 2 shows the link between the client PC and the server. The server has a component-based architecture, and is service and message oriented. A specific gateway has been built to ease communication with client PCs, providing secure access to data structures. It allows the transport of a description of classes from the server to the client PC. When a client PC sends data to the server, it communicates with the server gateway that transmits the request. The service directory is then queried to identify the appropriate service where the received information can be stored. All data transiting through the channel are formatted in XML.

What the new protocol needed was a client PC with the capability to work independently when a data connection was not available. This feature was necessary for those client PCs in rural or low-resource areas who had spotty data connections. The client PC layer (DataSync) was designed as an independent local app, yet it was able to synchronize with the server in real time when an Internet connection was available. Thus, DataSync featured a synchronization mechanism that allowed for the checking of an available Internet connection every 10 minutes.

##### Communication With Patients

The focus of the interactions between patients and SMSaúde was to promote self-management and adherence to treatment. SMS text messages were sent to patients according to the following scheme: (1) *appointment reminders* were sent 15, 7, and 2 days before their scheduled outpatient appointments, and 4 and/or 7 days after scheduled appointments if the patients had failed to show up in the health center, (2) *medication reminders* were sent monthly and every 2 months, (3) *motivational messages*, and (4) *educational messages* were both sent monthly. SMS text message types (2), (3), and (4) were scheduled to be sent on Monday, Wednesday, and Friday, respectively. Overall, SMS text messages were scheduled to be sent on weekdays.

A two-way information exchange protocol was needed to support this self-management model and address a major weakness of the old system. What the new protocol needed was the capability to respond automatically to patients’ text messages. We developed an algorithm that reads the content of the message sent by the patient, interprets it, and decides what message to use for autoreply. This algorithm relies on a database table of keywords. The keywords were generated based on the text messages received from patients during the initial system implementation tests, and on the medical doctors’ experience and knowledge of patients’ needs. The algorithm not only looks for keywords, but also tries to make sense of the entire SMS text message. This is a function that holds great promise because it can increase the “density” in treatment communication and reduce the marginal cost for information, but must be used with care as misunderstandings will decrease patients’ trust in the system.

##### Portal Interface

We developed a Web-based user interface to allow clinicians to view patient information, including personalized text messages, and appointment and medication data. The interface is shown in Multimedia Appendix 1. The main components of the portal are the Dashboard, Messages (Mensagens), Patient Data (Dados dos Pacientes), and Charts. The Dashboard was designed to provide an overview and some statistics (frequencies) of patient information, such as appointment data, and scheduled, incoming, and outgoing messages. The Messages component was designed to display outgoing, incoming, and scheduled messages. The Patient Data component was designed to display patients’ information and appointment data received from the client PC. The Charts component displays statistics of messages (outgoing, incoming, and scheduled) in the form of a pie chart, bar chart, histogram, and timeline chart.

###  Demonstration

The SMSaúde system was implemented in five health facilities: Machava 2, Matola 2, Matola 1, Ndlavela and Namaacha at Maputo province in Mozambique. The choice of sites was based on ARK’s strategy and consisted of making sure that the following resources existed: human resources that included at least one nurse, one medical doctor, one medical counsellor, one pharmacy technician, and one data entry clerk, as well as one computer and one GSM modem connected to the computer. These resources were necessary as the system required that information generated by different health workers be populated in the EMR.

###  Evaluation

**Figure 3:**
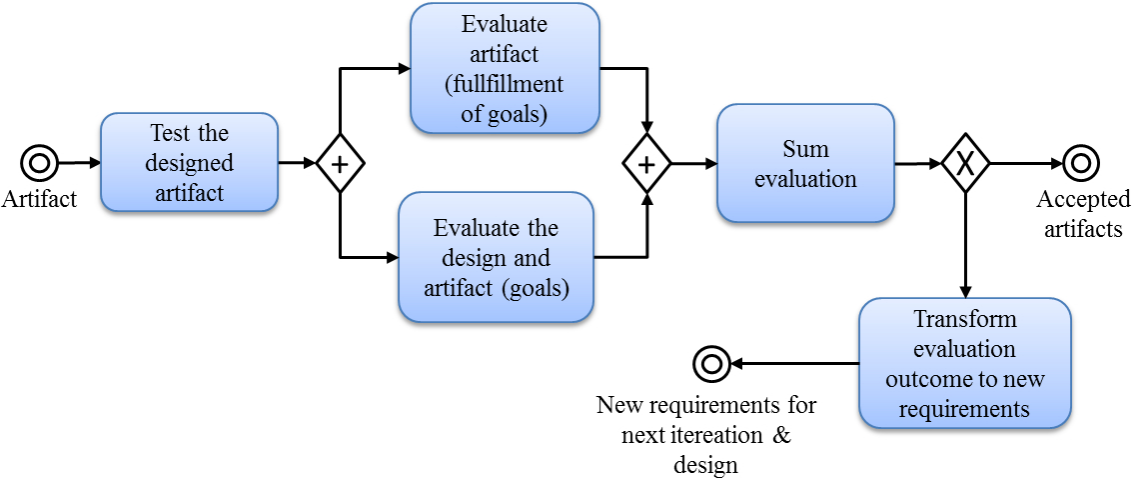
The process of evaluation of the SMSaúde artifact.

Testing and evaluation of the SMSaúde system—based on seven quality criteria—was done by collaborative teams composed of researchers, the development team, and two medical doctors. These quality attributes were functionality, completeness, consistency, accuracy, performance, reliability, and usability. The SMSaúde artifact was input into this process. Figure 3 shows the process of testing the SMSaúde artifact. The artifact was improved interactively and incrementally, in a collaborative manner, until the team perceived the artifact as “good enough” for its intended user and problem domain. The activity “test the designed artifact” involved carrying out the actual tests, for instance, examining the artifact in the intended problem domain. The information from the tests was then used in the evaluation activity and compared with the goals set. During the iterations, many features of SMSaúde were changed, removed, or added. During the testing and evaluation of the artifacts, the team listed ideas for improvement, for instance, new requirements for improved versions of the artifact.

The output of this process of evaluation was either artifacts classified as ready and approved by collaborative teams or artifacts that were classified as not accepted. If the artifacts were not accepted they needed to be further designed. Reflections and notifications from the evaluation activity provided additional input for a new iteration.

##  Discussion

### Principal Findings

This paper summarizes the processes of design, development, and implementation of a remote management system using mobile technologies to help patients with HIV and TB to improve self-management of their treatment. The system comprises an application layer for the capture and delivery of patient information, including date of next appointment and next date of collecting and taking medication, to a remote server by means of a GSM modem. The server generates personalized appointment reminders, medication reminders, as well as educational and motivational messages and sends them to patients. Health care professionals, technical experts, and an interdisciplinary group of researchers were involved in the design, development, and implementation processes to enhance the utility and functionality of the system.

Our research endeavors to design, develop, and implement a tool to empower patients in self-managing their HIV and TB and in their interactions with health care providers. Mobile phones are available and are practical, low-cost tools that may facilitate and support patient empowerment and involvement in their management of HIV and TB. Our literature study showed that there is evidence that mobile phone text messaging provides an effective communication mechanism between health care providers and patients, provided it is implemented well. It has been shown that mHealth interventions have the potential to improve treatment and motivate patients to adhere to treatment, thus making better use of health care resources [33]. However, a major challenge for health care lies in transforming traditional patient-health care provider relationships in a way that the patient may take a more active and equal role in planning and decision making regarding his/her care and treatment, in line with person-centered approaches [34,35].

Design of a system for treatment of specific diseases like HIV/AIDS and TB, based on mobile phone text message technology, includes careful attention to not only the theoretical underpinning of the strategic intervention and associated content, but also the requirements for optimal design, including how each part of the system has to be placed together. Crucial parts of a successful text messaging intervention include how messages are tailored, how participants may be directed to different content based on their situation, and the intensity of the communication. The system must also include cost-effective mechanisms so as to minimize costs of both technologies involved (including costs for GSM network communication and GSM modems with its data plan) and of sending messages to patients. Other costs include having people entering data frequently.

Although the system was designed specifically for HIV and TB, this development strategy can be generalized to other text messaging-based interventions, maybe with a special focus in settings with limited resources. Certainly, addressing all requirements is important. However, they come with different urgency levels. While, for example, *data* and *connectivity* are basic for making the system work and some agreement on *message content* is crucial to make it at all happen, *privacy and data security* and *system scalability* should be developed with some care. Trust, usefulness, and usability are crucial issues for both patients and professional users, and it is important that features installed also work properly and meet privacy standards. Therefore, it is better to move slowly in making the system more advanced and, at the same time, making sure to have users on board.

###  Conclusions

This study demonstrated that it is possible to design, develop, and implement an integrated remote patient management system using the mobile phone’s text message feature to communicate with patients. The study shows that it is necessary to address the following six different requirements: data collection, telecommunication costs, privacy and data security, the content of text messages, connectivity, and system scalability.

###  Next Steps

We plan to perform an evaluation of the system, including a satisfaction survey of the health professionals and patients who used it. Additionally, we plan to perform a cost-benefit analysis.

## Multimedia Appendix 1

The SMSaúde portal. The table displays outgoing messages with their phone numbers and dates sent.

## Abbreviations

ARK: Absolute Return for Kids
ART: antiretroviral therapy
CIBS FM&HCM: Institutional Bioethics Committee on Health of Faculty of Medicine and Maputo Central Hospital
DSR: design science research
DSRM: design science research methodology
EMR: electronic medical records
EPTS: electronic patient tracking system
GSM: Global System for Mobile communications
IT: information technology
M-HELP: mobile health examination launched on the phone
PC: personal computer
SMS: short message service
SOC: standard of care
TB: tuberculosis
WSDL: Web services description language
XML: extensible markup language
