# Supplementary figures and images for: SMSaúde: Design, Development, and Implementation of a Remote/Mobile Patient Management System to Improve Retention in Care for HIV/AIDS and Tuberculosis Patients
Source: JMIR Mhealth Uhealth. 2015 Mar 9;3(1):e26. doi: 10.2196/mhealth.3854 (PMC4376127; doi:10.2196/mhealth.3854)

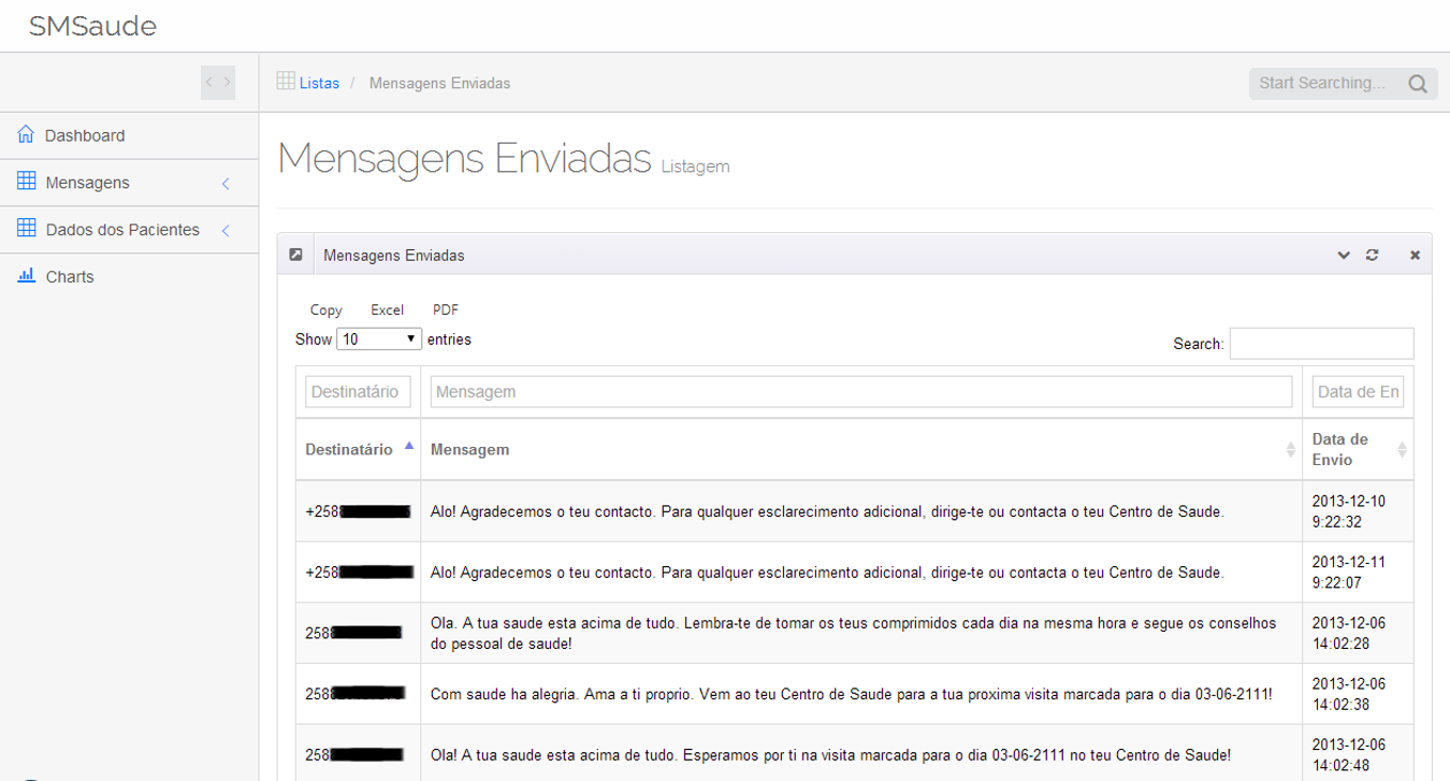

Supplement: Supplementary file 1 [file mhealth_v3i1e26_app1.jpg]
